# Mirror-Image Lesions in Sequential Relapses of AQP4-Positive Neuromyelitis Optica Spectrum Disorder

**DOI:** 10.3389/fneur.2020.00414

**Published:** 2020-05-12

**Authors:** Ryan T. Muir, Aditya Bharatha, Dalia Rotstein

**Affiliations:** ^1^Division of Neurology, Department of Medicine, University of Toronto, Toronto, ON, Canada; ^2^Division of Neuroradiology, Department of Radiology, St. Michael's Hospital, University of Toronto, Toronto, ON, Canada

**Keywords:** neuromyelitis optic spectrum disorder, MRI, astrocytopathy, blood brain barrier (BBB), aquaporin (AQP)-4

## Abstract

A 25 year-old Nigerian woman with aquaporin-4 antibody-positive neuromyelitis optica spectrum disorder (NMOSD) presented with a 6 week history of nausea, vomiting, and refractory hiccups; as well as progressive lower extremity sensory loss, weakness, saddle anesthesia, and urinary incontinence. She had experienced her first NMOSD relapse seven years prior with bilateral lower extremity weakness and area postrema syndrome. After pulse steroids and plasma exchange she made a complete neurologic recovery and was started on azathioprine. An initial aquaporin-4 (AQP4) antibody ELISA test was positive, but three subsequent tests were negative and repeat MRI brain showed resolution of T2/FLAIR signal abnormalities with the exception of a right thalamic lesion and a left medullary lesion. Azathioprine was discontinued after 1 year and she was lost to follow-up. With her second relapse, she had new lesions in her left thalamus and right medulla—a mirror image of the thalamic and medullary lesions associated with her first relapse. In addition, an MRI spine demonstrated a new longitudinally extensive transverse myelitis from T7 to L1 with edematous expansion of the cord. Her serum AQP4 antibody test using a cell-based assay was strongly positive. NMOSD lesions are typically associated with brain regions with high density of the AQP4 channel. These areas include optic nerves, hypothalamus, and the diencephalic and brainstem tissues that surround the cerebral aqueduct and third and fourth ventricles. Previous studies have demonstrated that those with relapsing NMOSD have a predilection for recurrence in the same neuroanatomical region as their first episode. We hypothesize, using data from prior pathologic and epidemiologic studies, that mirror image lesions, where the same anatomic sites are affected on the contralateral side of the brain or spinal cord, may appear in subsequent attacks due to (i) areas of high remaining AQP4 density and/or (ii) local compromise of astrocyte or blood-brain barrier (BBB) function that persists after the initial inciting attack.

## Background

Neuromyelitis Optica Spectrum Disorder (NMOSD) is a relapsing inflammatory disease of the central nervous system (CNS). The past few decades have witnessed a rapid evolution in the understanding of the clinical and radiographic manifestations as well as the underlying pathophysiologic mechanisms of NMOSD. NMOSD, previously known as Devic's disease, was first described in the late 19th century as a monophasic illness characterized by optic neuritis and myelitis (1). However, more recently, the discovery of the pathogenic aquaporin-4 (AQP4) antibody has led to an appreciation of the diverse phenotypic expression of this relapsing disease.

Neuroimaging studies have characterized lesion localization and features that help distinguish NMOSD from Multiple Sclerosis (MS). For example, in MS, spinal cord attacks are associated with short segment lesions with partial, predominantly dorsal cord involvement, whereas in NMOSD lesions are typically longitudinally extensive, spanning ≥ 3 vertebral bodies in length, and often have complete transverse involvement. For many years, brain lesions were considered atypical of NMOSD, but it is now recognized that they occur in about half of those with NMOSD. In one study, 18.1% had brainstem periventricular/periaqueductal lesions, 32.7% had periependymal lesions along the lateral ventricles, 3.4% had large hemispheric lesions, 6.0% diencephalic lesions, and 4.3% corticospinal tract lesions (2). In contrast, ovoid lesions adjacent to the body of the lateral ventricle as well as Dawson's finger lesions affecting the corpus callosum are commonly observed in MS, and rarely observed in NMOSD (2, 3). The presence of (i) periependymal lesions along lateral ventricles and (ii) longitudinally extensive transverse myelitis (LETM), coupled with the absence of juxtacortical/cortical lesions, periventricular lesions, and Dawson's fingers was 92% sensitive and 91% specific for NMOSD (2). Furthermore, diencephalic lesions in one study were not present in any case of MS and were therefore 100% specific to NMOSD (2). In another NMOSD study, patients with brain lesions in regions of high AQP4 expression that were also considered to be classic brain lesions for NMOSD experienced more extensive myelitis compared to those without (4).

The brain and spinal cord regions typically affected in NMOSD and visualized on MRI have been shown to have the greatest AQP4 channel density (5). The AQP4 channel is the predominant water channel in the brain and has an important role in the development and homeostatic regulation of the interfaces between brain and blood, as well as between brain and cerebrospinal fluid (CSF) (5). Immunohistochemistry studies highlight an abundant concentration of the AQP4 channel at: astrocytic end feet of the blood-brain barrier (BBB); the glial lamellae of the supraoptic nucleus of the hypothalamus, and the basolateral membranes of ependymal cells along periventricular and periaqueductal areas (5–7). AQP4 channels have also been described in the amygdala, midbrain raphe nuclei, reticular formation, red nucleus, and tegmentum of the pons (4). In the spinal cord, AQP4 channels are present to a greater extent in the central gray matter compared to white matter (7). Pathologic analysis of those with NMOSD has revealed a stage independent and targeted loss of AQP4 immunostaining from early active lesions right through to chronic lesions (7). In early active inflammatory perivascular lesions, in addition to AQP4 channel loss, there is vasculocentric immune complement activation and deposition which may drive astrocytic dysfunction and necrosis (7, 8).

These observations beg the question of whether individual variability in regions of greatest AQP4 density in the CNS may explain the regional predilection for subsequent relapses in NMOSD. In this report, we highlight a case of a patient with AQP4-IgG positive NMOSD with a second relapse affecting regions known to have high AQP4 density and mirroring the lesion locations of the first attack.

## Case

**Figure 1:**
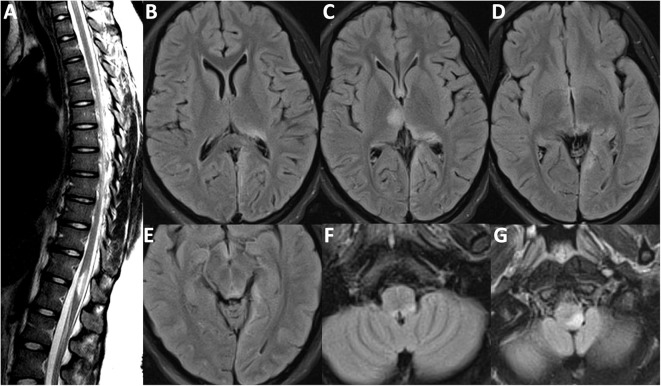
MRI Brain-FLAIR sequence from first attack in 2012. **(A)** Short segment transverse myelitis at T11/T12 vertebral segment with mild cord expansion. Lesions depicted affecting the **(B)** left posterior thalamus and the subcortical/juxtacortical white matter of the left medial occipital lobe; **(C)** right anterior thalamus and pulvinar region of the left thalamus; **(D)** highlighting left medial occipital lobe involvement; **(E)** periaqueductal gray matter hyperintensity; **(F)** left dorsal medullary hyperintensity also depicted in **(G)**.

Our patient, originally from Nigeria, first presented to an outside community hospital in 2012, when she was 17 years of age, after experiencing a witnessed generalized tonic-clonic seizure in the context of a 3 week history of progressive headaches, fever, nausea, refractory hiccups, and neck-stiffness, but without any cognitive or behavioral aberrancies. She had a lumbar puncture performed which revealed a white blood cell count of 250 cells/mL (91% lymphocytes), but bacterial culture and viral PCR studies were negative. Oligoclonal bands were not sent. She was initially started on acyclovir for presumed aseptic meningitis, however, she began to develop bilateral leg weakness and gait instability while in hospital. A brain MRI demonstrated T2/FLAIR signal hyperintensities in the right anterior thalamus, left posterior thalamus, left medial occipital lobe, and left dorsal medulla. A spine MRI revealed a short segment transverse myelitis at the T10/T11 vertebral levels. These images are depicted in Figure 1. Post-gadolinium T1 sequences did not reveal evidence of enhancement although, notably, the MRI was acquired after her course of intravenous methylprednisolone one gram daily for 5 days, followed by 100 mg daily of prednisone orally. Infectious causes were excluded prior to initiation of methylprednisolone. She had no improvement in her leg weakness. She was then transferred to our institution for consideration of plasma exchange. Repeated MRI brain and spine were unchanged and she received plasma exchange for 5 days under the presumption that she either had a demyelinating illness or Acute Disseminated Encephalomyelitis (ADEM). After seven cycles of plasma exchange, she gradually improved and slowly regained her ability to ambulate independently. An initial AQP4 antibody ELISA test performed at the outside hospital was verbally reported as positive (titer unavailable). She was subsequently started on azathioprine 150 mg daily. She made an excellent recovery and eventually resumed full activities as a student.

During the following year, three subsequent AQP4 antibody ELISA tests returned negative and repeat MRI demonstrated resolution of her occipital lesions and improvement of diencephalic lesions. With three negative ELISA tests and only a verbally reported positive initial AQP4 antibody ELISA test, her treating neurologist came to favor a diagnosis of ADEM and discontinued azathioprine a year after the initial presentation without further use of immunosuppressive or immunomodulatory agents.

Seven years later, at the age of 25 years, she presented to hospital with 6 weeks of progressive neurologic decline, which started with hiccups, nausea, and vomiting. Two weeks after onset, while still experiencing hiccups and nausea, she developed tingling and burning of the complete right leg, as well as lumbar and buttock pain. Four weeks after her initial symptoms started, she developed right leg weakness and subsequently left leg weakness. By the 5th and 6th weeks after onset she was unable to ambulate independently and developed saddle anesthesia, urinary incontinence. She presented to the emergency room for urgent medical attention.

At presentation, refractory hiccups were noted and bladder scan indicated retention of 750 mL of urine. Mental status was appropriate. Cranial nerve examination was unremarkable with preserved visual acuity, no evidence of red color desaturation, relative afferent pupillary defect, internuclear ophthalmoplegia, or nystagmus. Fundoscopy did not reveal any optic disc pallor or atrophy. Motor exam revealed full power in the upper extremities, but she had grade four-weakness in a pyramidal pattern in the bilateral lower extremities. While reflexes were preserved in the upper extremities, they were absent in the lower extremities. There was an extensor plantar response on the right and equivocal response on the left. She had near-absent pinprick and vibration sensation in the bilateral lower extremities with a discernable spinal level at the umbilicus. Sensation was entirely preserved in the upper extremities.

**Figure 2:**
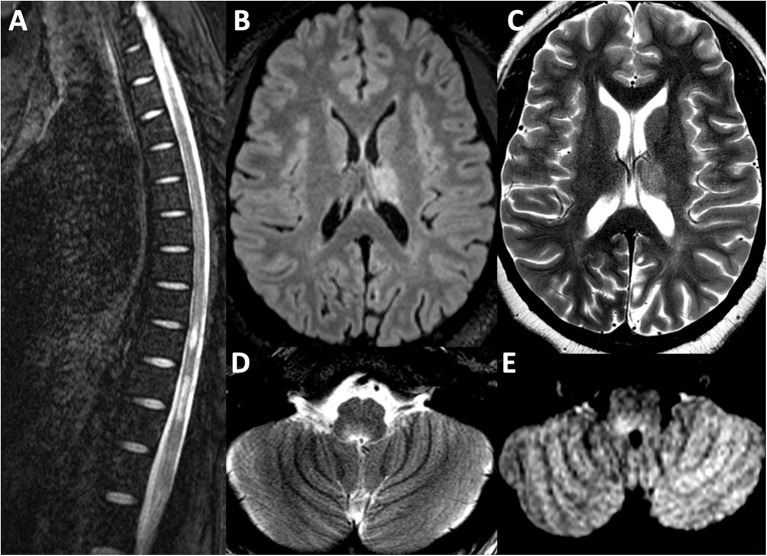
MRI Brain sequence from the second attack in 2019. **(A)** T2 weighted sequence depicting longitudinally extensive transverse myelitis from T7 to L1, associated with mild cord expansion. Lesions depicted affecting the left thalamus are depicted in the T2-FLAIR sequence in **(B)** and the T2 weighted sequence in **(C)**. A new right ponto-medullary lesion is visualized on the T2 weighted sequence in **(D)** and T2-FLAIR sequence in **(E)**.

Her MRI brain demonstrated new FLAIR hyperintense lesions in her left thalamus and right dorsal medulla. Old lesions were visualized in the right thalamus and left dorsal medulla. MRI of the whole spine revealed a longitudinally extensive T2 hyperintensity in the thoracic and lumbar regions from T7 to L1 with associated edematous expansion of the cord. These images are depicted in Figure 2. Unfortunately, gadolinium-enhanced sequences were not performed. Her serum AQP4-IgG test using a cell-based assay returned strongly positive with a titer of 4+. Myelin oligodendrocyte glycoprotein antibody was negative. CSF testing was not repeated. After her MRI, she received a 5 day course of intravenous methylprednisolone at one gram daily without clinical improvement. Plasma exchange was then initiated for seven cycles and over the course of 2 weeks the patient regained leg strength and sensation in her lower extremities, and, furthermore, her saddle anesthesia, urinary retention, and incontinence improved as well. She was started on mycophenolate 1 g twice daily in hospital and was ultimately discharged to a neuro-rehabilitation center. Rituximab, an anti-CD20 monoclonal antibody therapy, was also considered for maintenance therapy, but access to and funding for rituximab for treatment of NMOSD are severely restricted in Ontario, Canada. Three months after her discharge she was seen in follow up with repeat spine and brain MRI which demonstrated interval stability of her CNS disease.

## Discussion

The typical locations affected by the inflammatory lesions of NMOSD have a high density of the AQP4 channel (9). These areas include the optic nerves, hypothalamus, and the astrocytic end feet that abut capillaries and pia in the brain—namely diencephalic and brainstem tissues that surround the cerebral aqueduct, third, and fourth ventricles (1, 2, 5). There is also an abundance of AQP4 channels in the gray matter of the spinal cord.

In our report, we describe a patient with relapsing NMOSD whose inflammatory lesions mirrored those of her first attack. Mirror lesions occur in the same anatomic region on the contralateral side of the brain or spinal cord. Often one lesion may be directly adjacent to the other. Other times, as with the contralateral thalamic lesions observed in this case, lesions may surround the same ventricular space. Our patient's brain MRI demonstrated new lesions in the previously unaffected left thalamus and right dorsal medulla—creating a mirror image of her first diencephalic and brainstem lesions.

In a study of 164 patients with relapsing NMOSD, recurrent attacks were more likely to present with clinical features localizing to the same anatomic site(s) as the initial episode (10). This observation encompasses attacks that occurred in the same anatomic region on the contralateral side of the brain or spinal cord. For example, an increased odds of a second attack occurring in the initial event location were seen in all localizations, with the greatest odds of regional recurrence noted in the brain and brainstem (10). Furthermore, with a first attack of myelitis, there was a statistically significant 74% reduced odds of a second attack presenting as optic neuritis. It is interesting to note that our patient, despite extensive diencephalic, brainstem, and spinal cord lesions had never experienced an episode of optic neuritis. Even among those with established NMOSD there may be regional variability in AQP4 density and vulnerability to AQP4 antibodies associated with a predilection for relapses at anatomic sites similar to the initial event.

In addition to greater density of AQP4 in affected regions, a second theory to explain this regional predilection is that astrocytes and/or the BBB could be compromised at the sites of previous inflammation, making these regions or adjacent regions more likely to be affected in subsequent relapses. It is unknown exactly how AQP4-IgG initially crosses the BBB and gains access to the AQP4 antigen. Complement dependent and/or antibody mediated astrocyte cytotoxicity in NMOSD may further compromise the integrity of the BBB or lining of circumventricular organs leading to a kindling effect (7). Pathologic studies in NMOSD have demonstrated, consistently, the persistent loss of AQP4 immunohistochemistry within inflammatory lesions at all stages, but in the surrounding peri-plaque white matter there is a similar degree of AQP4 staining as normal regionally matched controls (8). This is in contrast to MS where AQP4 immunostaining is temporarily diminished in actively demyelinating lesions, but in remyelinating MS lesions, AQP4 immunoreactivity is diffusely increased in active astrocytes (8). In NMOSD, a glial astrocytopathy, AQP4 immunoreactivity remains low even during the recovery phase. Furthermore, the loss of glial repair mechanisms in NMOSD may confer an additional propensity for recurrence in adjacent regions or periventricular locations (7). It is possible that recently observed clusters of attacks soon after NMOSD presentation, with iterative attacks presenting with similar clinical manifestations, could be a reflection of regionally compromised repair mechanisms (11). However, future studies would be needed to test this hypothesis.

Interestingly, more extensive myelitis has been observed in patients with NMOSD whose lesions occur with greater frequency in typical AQP4 dense brain regions compared to those with NMOSD without AQP4 regionally typical lesions (4). This finding suggests that the topography of lesions in NMOSD may have pathophysiologic significance on the clinical course of NMOSD. In this case, our patient had a large LETM spanning seven vertebral segments. One can postulate whether the propensity of our patient's lesions to occur and recur in areas with greatest AQP4 channel density may have some relationship to the magnitude of her LETM.

With our patient's initial presentation in 2012, the diagnosis of ADEM was strongly considered. Her initial AQP4 antibody ELISA test was apparently positive, which would argue against ADEM, but three subsequent tests were negative which led the treating neurologist at the time to believe that the initial ELISA could have been a false verbal report or false positive test result. Particularly in the past when AQP4 and MOG serologic testing was less reliable, it could be difficult at times to distinguish the first episode of NMOSD from ADEM, as ADEM can also present with LETM, large hemispheric lesions, as well as diencephalic and brainstem lesions. In one study, thalamic and internal capsule involvement were found to occur more frequently in ADEM than in NMOSD (12). Our patient's initial presentation with thalamic lesions, meningismus, fevers, and a seizure led the treating neurologist to favor a diagnosis of ADEM after the first attack. This case highlights overlapping clinical features in ADEM and NMOSD, particularly in pediatric cohorts, and emphasizes the need for repeat AQP4 cell-based assay testing when there is a strong index of clinical suspicion for NMOSD.

The implications of this report are limited by the fact that this is a single case. Although we did not find any other cases in the literature of mirror-image lesions reported in association with AQP4 positive NMOSD, a recent international study supported the tendency of sequential NMO relapses to have similar localizing features (13). One reason why we may be the first to report this phenomenon is that it is difficult to discern mirror-image lesions in NMOSD in locations other than the brain. Myelitis and optic neuritis are much more common relapse types in NMOSD. Spinal cord lesions are often bilateral obviating the opportunity to observe mirror-image lesions. With respect to optic neuritis, bilateral simultaneous optic neuritis is a hallmark feature of NMOSD, and itself could be considered an example of mirror-image lesions.

In summary, our report highlights a case of relapsing NMOSD with recurrent lesions occurring in the same region, but contralateral to the lesions implicated in the first attack. Mirror-image lesions may be due to effects of the pathogenic antibody on areas of high remaining AQP4 density and individual variability in the most dense AQP4 regions. We hypothesize, using data from previous pathologic and epidemiologic studies, that regions mirroring the prior attack site may be vulnerable to recurrent attacks due to (i) patterns of high AQP4 antigen density in the CNS and/or (ii) local compromise of astrocyte and/or BBB functions.

## Data Availability Statement

The raw data supporting the conclusions of this article will be made available by the authors, without undue reservation, to any qualified researcher.

## Ethics Statement

Ethical review and approval was not required for the study on human participants in accordance with the local legislation and institutional requirements. The patients/participants provided their written informed consent for the publication of this case report.

## Author Contributions

RM: study conception and design, manuscript preparation. AB: study conception and design, manuscript preparation, and neuroimaging figure preparation. DR: study conception and design, manuscript preparation, acquisition of data, and final approval of manuscript.

## Conflict of Interest

DR has received research support from the Multiple Sclerosis Society of Canada, CMSC, and Roche Canada. She has served as a consultant or speaker for Alexion, Biogen, EMD Serono, Novartis, Roche, and Sanofi Aventis. The remaining authors declare that the research was conducted in the absence of any commercial or financial relationships that could be construed as a potential conflict of interest.
